# EHMT2 promotes the pathogenesis of hepatocellular carcinoma by epigenetically silencing *APC* expression

**DOI:** 10.1186/s13578-021-00663-9

**Published:** 2021-08-03

**Authors:** Yuan Guo, Yan-Rong Zhao, Huan Liu, Yang Xin, Jian-Zhi Yu, Yun-Jin Zang, Qing-guo Xu

**Affiliations:** 1 Liver Disease Center, The Affiliated Hospital of Qingdao University, 59 Haier Blvd, Qingdao, 266000 Shandong China; 2 Organ Transplantation Center, The Affiliated Hospital of Qingdao University, 59 Haier Blvd, Qingdao, 266000 Shandong China; 3 Lead Contact, The Affiliated Hospital of Qingdao University, 59 Haier Blvd, Qingdao, 266000 Shandong China

**Keywords:** Hepatocellular carcinoma, EHMT2, Wnt–β-catenin pathway, APC, UNC0642

## Abstract

**Background:**

Hepatocellular carcinoma (HCC), the second leading cause of cancer death worldwide, alone accounts for over half (466,100) of new cancer cases and 422,100 deaths based on the average year incidence rates of 2009 to 2011 in China. Due to unclear and complex underlying mechanisms for HCC development, effective therapy for HCC is still unavailable. The Wnt–β-catenin pathway is a critical contributor of HCC pathogenesis: 40–70% of HCCs from patients harbor the nuclear accumulation of β-catenin protein. However, the mechanisms for β-catenin activation are not fully understood.

**Methods:**

The deletion of EHMT2 in Hep3B and Huh1 cells was achieved by transiently transfecting cells with pX459 plasmids, which carry EHMT2 specific small guide RNA (sgRNA) sequences for Cas9 protein. All experiments were performed in triplicate and repeated more than three times.

**Results:**

In the present study, we observed that *EHMT2* (but not *EHMT1*) mRNA and protein levels were significantly elevated in HCC compared with normal controls. Next, the results of Ki67 staining, as well as MTT, soft-agar and xenograft assays, in wild-type and *EHMT2*^−/−^ Hep3B and Huh1 cancer stem cells collectively revealed that the elevation of EHMT2 expression is required for the tumorigenesis of HCC. Meanwhile, we found that elevated EHMT2 expression contributes to the activation of Wnt–β-catenin signaling: deletion of *EHMT2* in Hep3B or Huh1 cells promoted the cytoplasmic localization of β-catenin and restrained the expression of Wnt–β-catenin signaling targets such as *Myc*, *CCND1*, *MMP-7*, etc. We demonstrated that EMHT2 directly mediates the H3K9me2 methylation of the *APC* promoter to epigenetically silence its expression. More intriguingly, our findings also showed that UNC0642, a specific inhibitor of EHMT2, exhibits anti-tumorigenesis effects in HCC both in vitro and in vivo, which were largely abolished by deletion of *EHMT2* or overexpression of *APC* in Hep3B and Huh1 cells.

**Conclusion:**

Altogether, our observations emphasize that the EHMT2–APC axis is a critical contributor to Wnt–β-catenin pathway activation in HCC, and UNC0642 may be a potential candidate for target drug treatment of HCC.

**Supplementary Information:**

The online version contains supplementary material available at 10.1186/s13578-021-00663-9.

## Introduction

Hepatocellular carcinoma (HCC), the second leading cause of cancer death worldwide, alone accounts for over half (466,100) of the new cases and 422,100 deaths in recent years in China [1, 2]. Currently, risk factors including hepatitis B or C viral infections, alcohol-related cirrhosis, and non-alcoholic steatohepatitis have been frequently reported to mainly cause HCC [3–5]. In China, due to the difficulties in early diagnosis, HCC also has the poorest survival with an age-standardized 5-year relative survival of only 10.1% [6, 7]. Moreover, HCC is a heterogeneous disease with highly heterogeneous risk factors, environmental or genetic susceptibilities, morphological diversity, signaling network disorders, and microenvironmental discrepancies, which largely limit the prediction of disease progress, molecular classification, and targeted therapy [8, 9]. However, the underlying mechanisms for HCC development are still exclusive. In treatment, only sorafenib, an anti-angiogenic and MAP kinase inhibitor, has been shown to improve the prognosis outcome of HCC patients [10]. To date, although multiple clinical trials with agents targeting FGF, VEGF, PDGF, EGF, IGF, mTOR, and TGFβ signaling pathways have been carried out, the effective therapy for HCC is still unavailable.

The Wnt–β-catenin pathway is a key molecular mechanism that involves the processes of embryonic development and tissue homeostasis [11, 12]. In rest status, β-catenin (encoded by gene *CTNNB1*) exposes its armadillo domain repeats to bind its interactors including Axin1 and APC (adenomatous polyposis coli protein) as well as GSK3B for the cytoplasmic arrest and ubiquitination-mediated degradation by the proteasome [13]. When a Wnt ligand (such as Wnt3a) binds to a Frizzled (FZD) receptor, a seven-transmembrane domain protein at the cell membrane, the cytoplasmic sequestered β-catenin is released and translocates into the nucleus to interact with transcriptional factors of the TCF/LEF family to promote the expression of Wnt–β-catenin signaling targets including *Myc, CCND1, MMP-7*, etc. [11, 14, 15]. In the pathogenesis of HCC, the Wnt–β-catenin pathway is a critical contributor, evidenced by 40–70% of HCCs harbor nuclear accumulation of the β-catenin protein [14, 16, 17]. Specifically, activation of β-catenin during hepatocarcinogenesis is mainly mediated by *CTNNB1* gene mutation (in 8–30% tumors) or loss-of-function mutation of *APC* (in 1–3%)/*AXIN1* (in 8–15%) [18–20]. However, in some special cases without mutations of *CTNNB1, APC*, and *AXIN1*, the mechanisms of how the β-catenin in HCC is activated are not fully understood.

EHMT2 (also known as G9a), a nuclear histone lysine methyltransferase, specifically mono- and di-methylates 'lysine-9' of histone H3 (H3K9me1 and H3K9me2, respectively) in euchromatin [21, 22]. Generally, H3K9me in gene promoters epigenetically mediates gene transcription repression by recruiting heterochromatin components such as heterochromatin protein 1 (HP1) to the methylated histones [23]. Over the past decades, EHMT2 has been demonstrated to display fundamental functions in embryogenesis in genetic mouse models, for example, deletion of *EHMT2* in mice resulted in embryonic lethality [24, 25]. Recently, the roles of EHMT2 in cancers have been extensively explored by different groups. Overexpression of *EHMT2* has been observed in various cancers including esophageal squamous cell carcinoma, aggressive lung cancer, multiple myeloma, brain cancer, ovarian carcinoma, etc. [26–28]. More importantly, the high *EHMT2* expression was shown to be associated with poor outcomes of patients' survival [28–30]. The elevated *EHMT2* expression level is commonly correlated with higher methylation levels and suppression of important tumor suppressor genes expression, suggesting that the alteration is functional. In 2018, Zhang et al. reported that EHMT2 is upregulated in non-small cell lung cancer (NSCLC) to epigenetically restore APC2 functions and then silences the Wnt–β-catenin pathway [23]. In this study, we aimed to investigate the roles of EHMT2 in the pathogenesis of hepatocellular carcinomas and identify the underlying mechanisms.

## Methods and materials

### Patient specimens

In the present study, tumor and adjacent normal tissues were collected from 33 patients with liver hepatocellular carcinoma who were enrolled in the Affiliated Hospital of Qingdao University from Sept 2016 to Oct 2018. All the related pathological sections were identified by three independent oncologists. The fresh specimens were quickly frozen in liquid nitrogen for protein extraction and immersed into RNAlater™ Stabilization Solution (Cat: AM7020, ThermoFisher Scientific, Waltham, USA) for RNA extraction. The written informed consent forms were obtained from the patients or their families. All the protocols involved in our experiments were approved by the Ethics Committee of Qingdao University and study methodologies conformed to the standards set by the Declaration of Helsinki.

### Cell culture

Hep3B cells (Cat: HB-8064) were purchased from American Type Culture Collection (ATCC, Manassas, USA). Hep3B cells were cultured in ATCC-formulated Eagle's Minimum Essential Medium (Cat: 30-2003, Manassas, USA) plus 10% fetal bovine serum (FBS, Gibco, Rockville, USA) and 50 μg/ml penicillin/ streptomycin (P/S, Gibco, Rockville, USA). Huh1 (Cat: JCRB0199) cells were purchased from the Japanese Collection of Research Bioresources Cell Bank (JCRB, Osaka, Japan), and were cultured in Dulbecco's Modified Eagle Medium (DMEM, Gibco, Rockville, USA) plus 10% FBS and 50 μg/ml P/S. Cells were maintained in an incubator at 37 °C with 5% CO_2_. For transient transfection, PolyJet reagent (SL100688, Signagen, USA) was used following the manufacturer's instructions.

### Construction of KO cell lines

The deletion of *EHMT2* in Hep3B/Huh1 cells was achieved by transiently transfecting cells with pX459 plasmids, which carry EHMT2 specific small guide RNA (sgRNA) sequences for Cas9 protein. The plasmids were generated by directly ligating synthesized oligos with linearized pX459 vector by BbsI restriction enzyme (Cat: R0539V, New England Biolabs, Ipswich, USA). Three independent sgRNAs were designed based on NGG protospacer adjacent motif (PAM) sequences (https://zlab.bio/guide-design-resources). The related sgRNA sequences are listed as follows: KO-sgACSM3#1: 5′-CCT CGT GGC TCC TTG GCC CG-3′; KO-sgACSM3#2: 5′-CGT GGC TCC TTG GCC CGC GG-3′; KO-sgACSM3#3: 5′-CAA GGA GCC ACG AGG TGA GG-3′. After 36 h of transfection, the cells were selected using puromycin at a dose of 1.0 μg/ml (Cat: A1113803, ThermoFisher Scientific, Waltham, USA) for 1–2 days. When cells recovered from anti-biotin selection, the single-cell clones were sorted using a flow cytometer. About 30 days later, the grown-up single clones were picked up and were subjected to sequencing and Western blot identification.

### RNA extraction and real-time quantitative PCR RT-qPCR

Tissue or cell mRNAs were extracted using TaKaRa MiniBEST Universal RNA Extraction Kit (Cat: 9767, Tokyo, Japanese) following the manufacturer's instructions. cDNAs were synthesized using PrimeScript™ RT Master Mix (Cat: RR036B, Takara, Tokyo, Japanese). The specific mRNAs were quantified by RT-qPCR using TB Green Fast qPCR Mix (Cat: RR430B, Takara, Tokyo, Japanese) on the Thermal Cycler Dice Real-Time System III (Code No. TP950, Takara, Tokyo, Japanese). The EHMT2 levels were calculated by the 2^−△△CT^ method with ACTB mRNA as the internal control. The oligos used for RT-PCR were listed as follows. *EHMT2*: forward, 5′-TCC AAT GAC ACA TCT TCG CTG-3′; reverse, 5′-CTG ATG CGG TCA ATC TTG GG-3′. *ACTB*: forward, 5′-CAT GTA CGT TGC TAT CCA GGC-3′; reverse, 5′-CTC CTT AAT GTC ACG CAC GAT-3′. *Myc*: forward, 5′-GTC AAG AGG CGA ACA CAC AAC-3′; reverse, 5′-TTG GAC GGA CAG GAT GTA TGC-3′. *CCND1*: forward, 5′-CAA TGA CCC CGC ACG ATT TC-3′; reverse, 5′-CAT GGA GGG CGG ATT GGA A-3′. *MMP-7*: forward, 5′-ATG TGG AGT GCC AGA TGT TGC-3′; reverse, 5′-AGC AGT TCC CCA TAC AAC TTT C-3′.

### Western blot

Specifically, for the Western blot of nuclear proteins, the nuclei of cells were isolated using Nucleoprotein Extraction Kit (Cat: C500009, Sangon Biotech, Shanghai, China). After quantified with Pierce™ BCA Protein Assay Kit (Cat: 23225, ThermoFisher Scientific, Waltham, MA USA), equal protein samples were resolved in 10% SDS-PAGE gel. Then, the separated proteins were transferred onto nitrocellulose filter (NC) membrane. After blocked in 5% non-fat milk/PBS for 1 h at room temperature, the membranes were washed with TBST for 3 × 5 min and then incubated with primary antibody at 4ºC overnight. The next day, the membranes were washed with TBST for 3 × 5 min at room temperature, and then were probed with the HRP (peroxidase)-conjugated secondary antibody at room temperature for 1 h. Signals were detected using Pierce ECL Western Blotting Substrate (Cat: 32106, ThermoFisher Scientific, Waltham, MA USA) on Tanon 5200 multi-automatic image analysis system (Tanon, Shanghai, China). The protein bands were quantified using ImageJ software. Primary antibodies of EHMT2 (Cat: #3306), β-Actin (Cat; #3700), β-catenin (Cat: #8480) were purchased from Cell Signaling Technology (Danvers, USA). H3K9me2 (Cat: 49-1007) was purchased from ThermoFisher Scientific (Waltham, MA USA).

### Immunofluorescence staining

Hep3B and Huh1 cells were seeded onto Poly-lysine (P4707, Millipore Sigma, Darmstadt, Germany) coated cover glasses. 1–2 days later, cells were washed with PBS three times at room temperature and then fixed with 4% paraformaldehyde Fix Solution (Cat: E672002, Sangon, Shanghai, China) for 15 min. Next, the fixed cells were permeabilized with 0.2% Triton X-100 (Cat: E-IR-R122, Elabscience Biotechnology, Wuhan, China) for 10 min at room temperature. Sections were incubated with Ki67 (Cat: 27,309–1-AP, Proteintech, Rosemont, USA) or β-catenin (Cat: #8480, CST) primary antibodies at 4ºC overnight. The next day, sections were washed using PBS for 3 × 5 min at room temperature, and then incubated with 4',6-diamidino-2-phenylindole (DAPI) dye (Cat: D1306, ThermoFisher Scientific, Waltham, MA, USA) and fluorophore-conjugated secondary antibodies at room temperature for 2 h. Images were captured by the Leica microscope.

### Cell proliferation determination

Cell proliferation was determined by the MTT (Methylthiazolyldiphenyl-tetrazolium bromide) assays. MTT assay, based on the conversion of water-soluble MTT compound to an insoluble formazan product, was performed using a kit from Abcam (Cat: ab211091, Cambridge, USA) under the manufacturer's instruction. Briefly, 1.0 × 10^4^ cells were seeded onto 96-well plates at the indicated points including 0, 0.5, 1.0, 1.5, 2.0, 2.5, and 3.0 days. Next, the culture medium was replaced with serum-free media and MTT reagent (10 μl) when the cells of the final round seeding totally spread on the plates. After a 3-h incubation at 37 °C, MTT solvent was added to incubate for 15 min, and then all the plates were briefly mixed to ensure homogeneous distribution of color. Finally, the absorbance was measured at a 590 nm wave using a Synergy HTX microplate reader (BioTek, Beijing, China).

### Colony formation assay

The soft agar experiment was carried out as previously described [31]. Briefly, 1.0% agar and 2× related medium were evenly mixed at about 42ºC, and then cold to make the 0.5% base layer. Cells (1.0 × 10^3^) were thoroughly mixed with 0.35% top layer agar. After the top agar solid, the plates were placed in a 37 °C incubator for 3 weeks. Finally, the colony formation was determined by crystal violet staining.

### Xenograft assay

To determine the tumorigenesis of cells, Hep3B or Huh-1 cell lines were subjected to xenograft assay as previously described [32]. The nude mice BALB/c (n = 6 for each group) were purchased from Shanghai SLAC Laboratory Animal Co., Ltd (Shanghai, China). Cells (3.0 × 10^6^, in 150 μl sterile PBS) were subcutaneously injected into both sides of the animals. If necessary, the animals were treated with UNC0642 at 5 mg/kg via intraperitoneal injection with an interval of 3 days. Tumor volumes were recorded every 5 days by vernier caliper for 1 month.

### TCGA database analysis

Expression profile data of liver hepatocellular carcinoma (LIHC; tumor, n = 369; normal, n = 50) from The Cancer Genome Atlas (TCGA) and normal tissues (n = 160) from Genotype-Tissue Expression (GTEx) were analyzed by GEPIA online tool (http://gepia2.cancer-pku.cn/#index). The expression pattern of *EHMT1/2* was analyzed using a GTEx RNAseq datasheet for normal human tissues. The TPM was normalized and presented in log2-transformed values. In overall and disease-free survival analysis, GEPIA online tools were used, and the medium of *EHMT2* expression was used as a cutoff.

### Statistical analysis

All results were presented as mean ± SD, of which the duplication was specified in the related figure legends. GraphPad Prism 7 software (San Diego, USA) was used for statistical analysis. For two-group comparison, student's or paired *t*-test was used; for three or multiple group comparison, one- or two-way ANOVA was used, followed by post hoc Bonferroni multiple comparisons. A *p* < 0.05 was considered as statistical significance.

## Results

### High *EHMT2* expression is unfavorable for patients with HCC

**Fig. 1:**
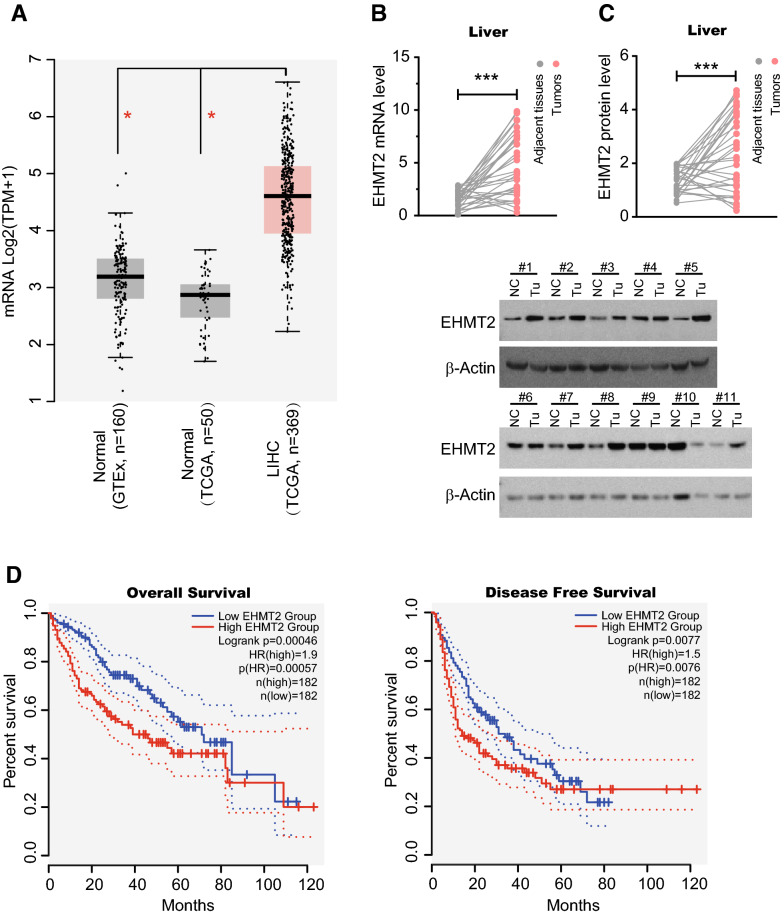
High *EHMT2* expression is unfavorable for patients with HCC. **A** *EHMT2* expression (mRNA) level in liver hepatocellular carcinoma (LIHC). Data from TCGA (tumor tissues, n = 369; normal tissues, n = 50) and GTEx (normal tissues, n = 160) database were analyzed by GEPIA online tool. TPM, transcripts per million reads. Line, medium; box, interquartile range (IQR); whiskers, non-outlier range; *p < 0.01. **B** mRNA level of *EHMT2* in human specimens was determined by RT-qPCR. n = 31, ***p < 0.001 by paired *t*-test. **C** the protein level of EHMT2 in human specimens was determined by Western blot. The bottom section shows the representative of Western blot results. Samples are the same as 1B. NC, adjacent normal controls; Tu, tumors. n = 31. ***p < 0.001 by paired *t*-test. **D** EHMT2-based overall and disease-free survival analysis of patients with HCC. In OS and DFS analysis, *EHMT2* expression data from TCGA were used, and the median of EHMT2 expression served as the cutoff. High *EHMT2*, expression level more than median; Low *EHMT2*, expression level less than the median. A log-rank test was used for the hypothesis test. OS, overall survival analysis; DFS, disease-free survival analysis

To explore the roles of *EHMT2* in HCC, we analyzed its expression level using the public data from TCGA and GTEx databases. Intriguingly, our results showed that the mRNA level of EHMT2 in HCC elevated significantly when compared with the normal controls from TCGA and GTEx (Fig. 1A). Consistently, these findings were confirmed by the expression data of clinical samples that we collected, indicated by *EHMT2* expression both in mRNA and protein levels were dramatically increased in liver tumors when compared with the paired adjacent normal tissues (Fig. 1B, C). These observations imply *EHMT2* expression level may exhibit clinical significance. To this end, we used the median of the *EHMT2* mRNA level as the cutoff value to carry out overall (OS) and disease-free survival (DFS) analysis with GEPIA online tools. Interestingly, we observed that high *EHMT2* expression predicted poor prognosis outcomes for patients with HCC both in OS (hazard ratio, HR = 1.9, *p* = 0.00057) and DFS (HR = 1.5, *p* = 0.0076) analysis (Fig. 1D). In 3 different molecular subtypes proposed by Chaisaingmongkol et al. [33], high *EHMT2* expression also exhibited unfavorable effects for prognosis outcome in OS analysis, especially in the iClust2/3 subtype (with a high HR > 2.7) (Additional file 1: Figure S1A). However, in DFS analysis based on *EHMT2* expression, no significant difference in prognosis outcome was observed in subtype iClust1/2 (Additional file 1: Figure S1B). In the iClust3 subtype, the survival percentage of the high-*EHMT2* group was significantly lower than the low-*EHMT2* group with an HR = 2.8 (*p* = 0.0043) in DFS analysis, showing a similar effect on OS of patients (Additional file 1: Figure S1B). Altogether, our data showed that the expression of *EHMT2* is elevated in HCC, which also indicates a poor clinical outcome.

### EHMT2 is required for the tumorigenesis of HCC

**Fig. 2:**
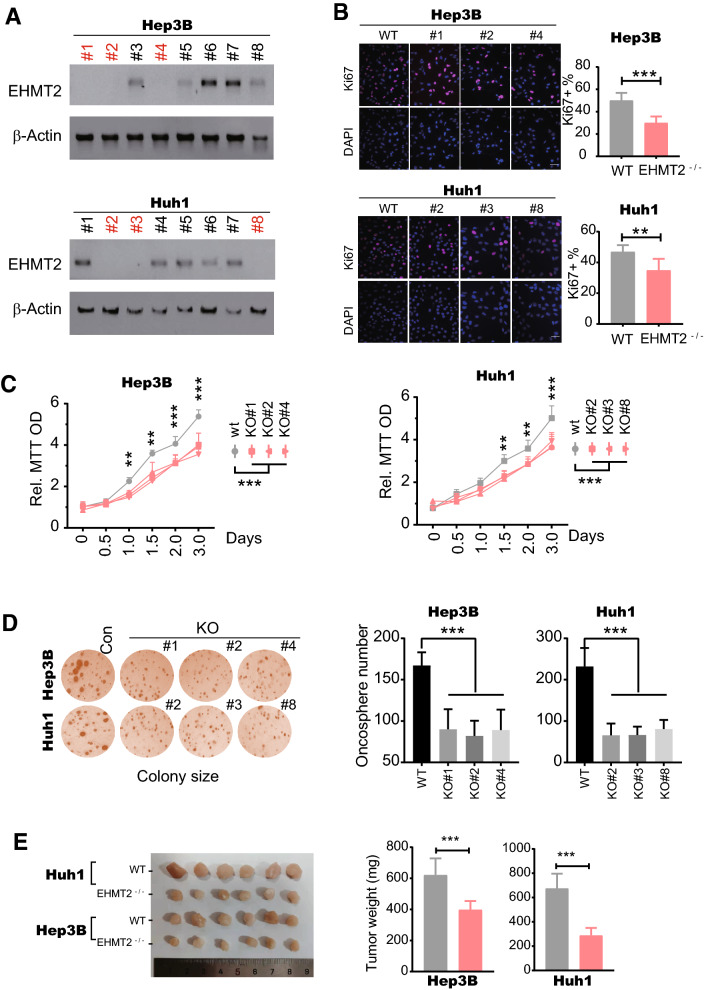
EHMT2 is required for the tumorigenesis of HCC. **A** The EHMT2 protein level in Hep3B and Huh1 cells was determined by Western blot. The red labels indicate the *EHMT2* double knockout (*EHMT2*^−/−^) single clones. n = 3. **B** Cell proliferation was determined by Ki67 staining. # indicates *EHMT2*^−/−^ single clones in 2A. Scale bar, 100 μm. n = 6 (mean ± SD); **p < 0.01 and ***p < 0.001 by student's *t*-test. **C** MTT assay determined the cell growth curve of Hep3B and Huh1 cells. EHMT2^−/−^ single clones were the same as 2A. n = 6 (mean ± SD); *p < 0.05, **p < 0.01 and ***p < 0.001 by two-way ANOVA followed by post hoc Bonferroni multiple comparisons. **D** Soft agar assay determined the anchorage-independent growth ability of Hep3B and Huh1 cell lines. *EHMT2*^−/−^ single clones were the same as 2A. The spheres with a diameter > 25 μm were considered tumorspheres. n = 6 (mean ± SD); ***p < 0.001 by one-way ANOVA. E. Final tumor weight of Hep3B and Huh1 in the xenograft assay. Three *EHMT2*^−/−^ single clones of Hep3B and Huh1 in 2A were pooled together for xenograft assay. n = 6 (mean ± SD); ***p < 0.001 by student's *t*-test

EHMT2, as an epigenetic "writer" (a lysine methyltransferase), catalyzes mono- and di-methylation of histone H3 lysine 9 (H3K9me1/2) and non-histone proteins, which has been demonstrated to implicate a variety of human diseases [34]. Therefore, we speculated that EHMT2 plays some roles in tumorigenesis of HCC. To this end, we constructed several *EHMT2* gene knockout cell lines in Hep3B and Huh1 using CRISPR/Cas9 technology. After Western blot identification, we obtained *EHMT2* double-deletion (*EHMT2*^−/−^) Hep3B and Huh1 single clones (Fig. 2A). In immunofluorescence staining of Ki67, the Ki67 positive percentage was slightly but significantly decreased in *EHMT2*^−/−^ Hep3B and Huh1 cells (Fig. 2B). These data indicate EHMT2 possesses a proliferation-enhancing function in HCC cells. Similarly, our MTT assay (determining cell proliferation) result also displayed that wild-type Hep3B and Huh1 grew significantly faster than *EHMT2*^−/−^ ones since 1.0–1.5 days of seeding cells (Fig. 2C). Furthermore, we found the deletion of *EHMT2* effectively abated the anchorage-independent growth both in Hep3B and Huh1 cells, as shown in the decrease of colony size of *EHMT2*^−/−^ groups compared with wild type groups (Fig. 2D). These observations altogether demonstrate that EHMT2 possesses tumor-promoting function in HCC cells in vitro. Besides, we estimated EHMT2's tumor-promoting function in vivo using xenograft assay. In the xenograft assay, we observed that deletion of *EHMT2* in Hep3B and Huh1 led to tumor growth suppression (Additional file 1: Figure S2A). Consistently, our finding showed that, compared with wild-type cells, the final tumor weights of *EHMT2*^−/−^ Hep3B and Huh1 cells significantly reduced (Fig. 2E). These findings indicate that EHMT2 also promotes tumor growth in vivo. Taken together, the elevation of *EHMT2* expression is required for HCC tumorigenesis.

### EHMT2 inhibitor exhibits anti-tumorigenesis effects in HCC

**Fig. 3:**
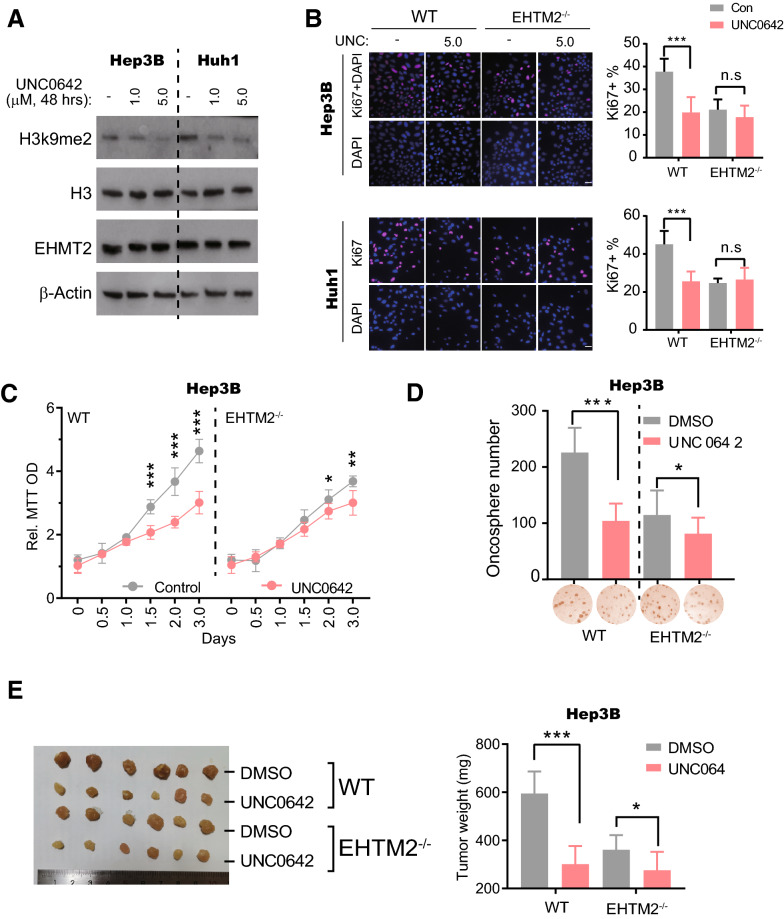
EHMT2 inhibitor exhibits anti-tumorigenesis effects in HCC. **A** The EHMT2 protein level in cells was determined by Western blot. Hep3B and Huh1 were treated with UNC0643 at the indicated conditions. n = 3. **B** Cell proliferation was determined by Ki67 staining. Three *EHMT2*^−/−^ single clones of Hep3B and Huh1 in 2A were pooled together for the experiment. Cells were treated with UNC0642 at a dose of 5.0 μM for 2 days. Scale bar, 100 μm. n = 6 (mean ± SD); n.s, no significance; ***p < 0.001 by student's *t*-test. **C** MTT assay determined the cell growth curve of Hep3B cells. Cells in 3B were treated with UNC0642 at a dose of 5.0 μM for 3 days. n = 6 (mean ± SD); *p < 0.05, ** *p* < 0.01 and ***p < 0.001 by two-way ANOVA followed by post hoc Bonferroni multiple comparisons. **D** Soft agar assay determined the anchorage-independent growth ability of Hep3B cell lines. Cells in 3B were treated with UNC0642 at a dose of 5.0 μM every 3 days. The spheres with a diameter > 25 μm were considered as tumorspheres. n = 6 (mean ± SD); *p < 0.05 and ***p < 0.001 by student's *t*-test. **E** Final tumor weight of Hep3B xenograft assay. Cells in 3B were used. UNC0642 treated animals with a dose of 5 mg/kg via intraperitoneal injection with an interval of 3 days. n = 6 (mean ± SD); *p < 0.05 and ***p < 0.001 by student's *t*-test

UNC0642, a well-known EHMT1/2 selective inhibitor, has been widely reported to inhibit the growth of multiple kinds of cancer cells including neuroblastoma, melanoma, bladder cancer, non-small cell lung cancer, etc. [31, 35–37]. However, whether UNC0642 is effective in HCCs is still exclusive. In our experiment, we also found that UNC0642 remarkably suppressed the enzymatic activity of EHMT2 without influencing its protein level, indicated by the reduction of H3K9me2 protein level after UNC0642 treatment in Hep3B and Huh1 cells (1.0 μM for 48 h) (Fig. 3A). In wild-type Hep3B and Huh1 cells, UNC0642 treatment dramatically decreased the percentage of Ki67 positive cells, which was significantly blocked by the deletion of *EHTM2* (Fig. 3B). Consistently, cell growth also was effectively suppressed by UNC0642 treatment on wild-type Hep3B cells (Fig. 3C). Of note, in *EHMT2*^−/−^ cells, UNC0642-induced alteration in cell growth was substantially decreased although not completely eliminated (Fig. 3C). In the soft agar assay, we also observed that the deletion of *EHMT2* partially blocked UNC0642-induced colony formation reduction in Hep3B cells (Fig. 3D). These findings suggested that UNC0642 suppresses the growth of HCC cells in an EHMT2-dependent manner in vitro. Moreover, in Hep3B and Huh1 xenograft assay, UNC0642 treatment on animals led to a prominent tumor growth inhibition (Fig. 3E and Additional file 1: Figure S3A). These effects of UNC0642 were strikingly eliminated by the loss function of EHMT2 (Fig. 3E and Additional file 1: Figure S3A). Collectively, UNC0642 exhibits anti-tumorigenesis effects in HCC both in vitro and in vivo by mainly targeting EHMT2.

### Elevation of EHMT2 promotes Wnt/β-catenin pathway activity

**Fig. 4:**
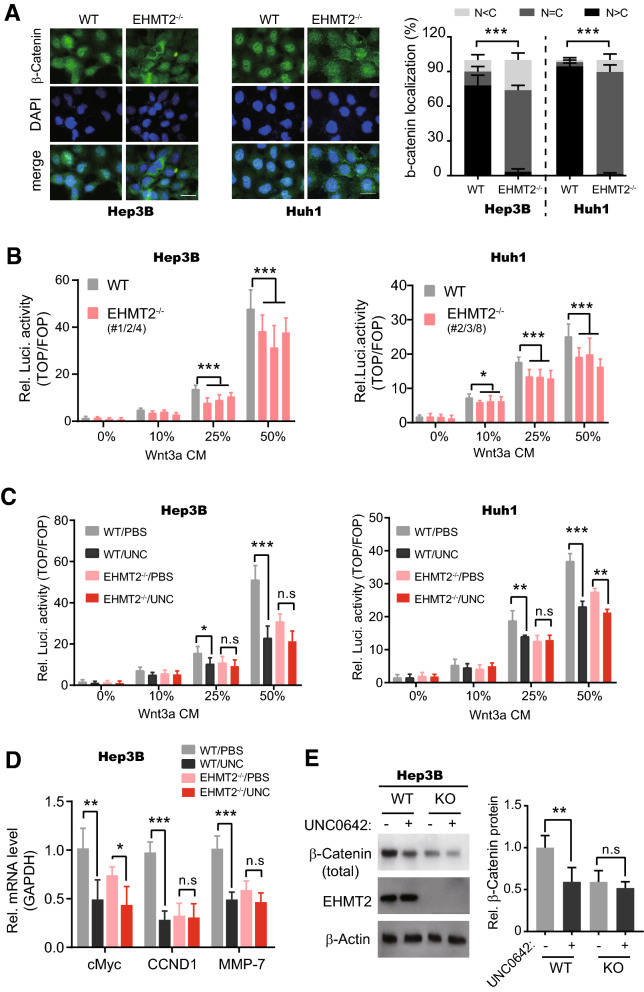
Elevation of EHMT2 promotes Wnt/β-catenin pathway activity. **A** The subcellular localization of β-catenin was determined by immunofluorescence staining. Scale bar, 50 μm. n = 6 (mean ± SD); ***p < 0.001 by student's *t*-test. **B** TOP/FOP luciferase showed Wnt–β-catenin signaling activity in Hep3B and Huh1 cells. Cells (wild type and *EHMT2*^−/−^ single clones in 2A) were treated with Wnt3a conditional medium (CM) at the indicated concentration. n = 6 (mean ± SD); *p < 0.05 and ***p < 0.001 by one-way ANOVA. **C** TOP/FOP luciferase showed Wnt–β-catenin signaling activity in Hep3B and Huh1 cells. Cells in 3B were pre-treated with UNC0642 (5.0 μM for 8 h) and then subjected to Wnt3a CM at the indicated concentration. n = 6 (mean ± SD); n.s, no significance, *p < 0.05 **p < 0.01 and ***p < 0.001 by student's *t*-test. **D** RT-qPCR showed Wnt–β-catenin signaling target gene expression in Hep3B cells. Cells in 3B were treated with 25% Wnt3a conditional medium. n = 6 (mean ± SD); n.s, no significance, *p < 0.05, **p < 0.01 and ***p < 0.001 by student's *t*-test. **E** The nuclear β-catenin protein level in cells was determined by Western blot. Cells and treatment were the same as 4D, and the nucleus of harvested cells was isolated. n = 6 (mean ± SD); **p < 0.01 and ***p < 0.001 by student's *t*-test

Previously, Zhang et al. reported that the overexpression of *EHMT2* in non-small cell lung cancer tissues epigenetically silenced tumor suppressor gene *APC2* to activate the Wnt–β-catenin signaling pathway, and EHMT2 inhibitor UNC0638 suppressed tumor growth [23]. In another line, UNC0642 treatment decreased the levels of histone H3K9me2 and Ki67 expression and increased apoptosis s in T24 and J82 cells (bladder cancer cells) [31]. In our experiments, we also found that β-catenin mainly localized in the nucleus (the activated form) both in Hep3B and Huh1 cells, and was expelled to the cytoplasm after deletion of *EHMT2* (Fig. 4A). These observations imply that EHMT2 is a maintainer of Wnt–β-catenin signaling activation in HCC cells. Therefore, we estimated Wnt–β-catenin signaling activity in cells with/without EHMT2 using TOP/FOP luciferase reporter, a widely used Wnt–β-catenin activity indicator system. Intriguingly, our results showed that, in Hep3B and Huh1 cells, Wnt–β-catenin activity (after Wnt3a conditional medium treatment) of three independent *EHMT2*^−/−^ single clones were substantially repressed when compared with that of wild type cells (Fig. 4B). Meanwhile, we observed that UNC0642 treatment efficiently suppressed Wnt3a-induced Wnt–β-catenin signaling activation both in wild-type Hep3B and Huh1 cells (Fig. 4C). Furthermore, our results revealed that around 61.8% of the effects of UNC0642 treatment were abolished by the deletion of *EHMT2* in Huh1 cells (Fig. 4C), suggesting UNC0642 influences the Wnt–β-catenin pathway activity mainly mediated by EHMT2. Consistently, the expression (in mRNA level) of Wnt–β-catenin pathway target genes including *Myc, CCND1, MMP-7*, etc.were suppressed by UNC0642 treatment on cells cultured in 25% Wnt3a conditional medium, which also was largely abolished by deletion of *EHMT2* in Hep3B cells (Fig. 4D). Also, the deletion of *EHMT2* obviously abrogated UNC0642-induced reduction of total and nuclear β-catenin protein level in Hep3B cultured in 25% Wnt3a conditional medium (Fig. 4E). Collectively, the elevation of *EHMT2* expression promotes Wnt–β-catenin pathway activity in different HCC cell lines.

### EHMT2 directly binds to the APC promoter to suppress its expression

**Fig. 5:**
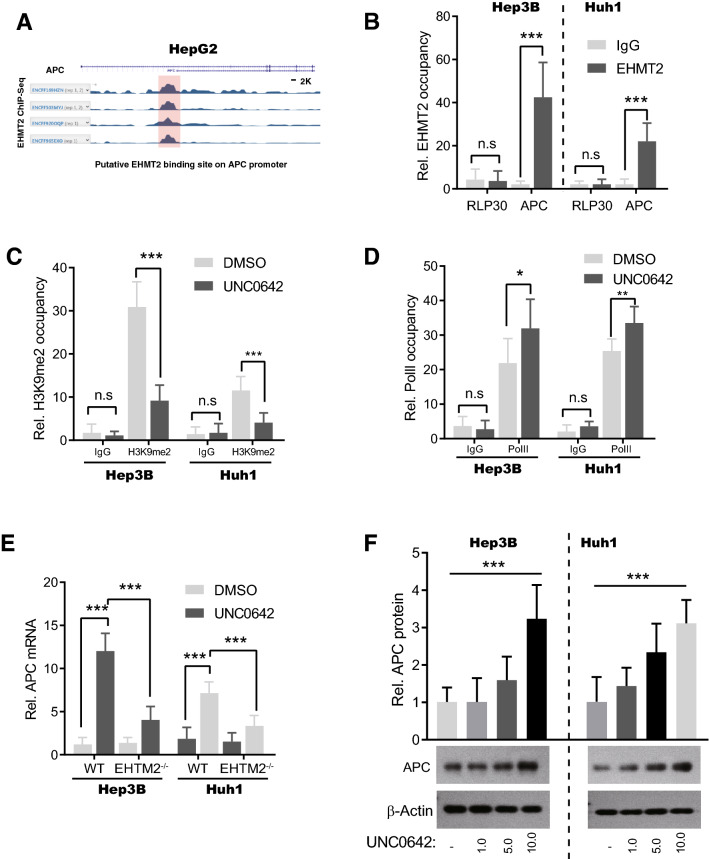
EHMT2 directly binds to the APC promoter to suppress its expression. **A** EHMT2 binding peak on the promoter of APC. Public ChIP-seq data using EHMH2 antibody in HepG2 cells are from the ENCODE database. The red rectangle indicates the binding signal peaks. **B** ChIP-PCR showed that EHMT2 directly binds to the *APC* promoter. 1.0 × 10^7^ Hep3B or Huhl cells were subjected to ChIP using the EHMT2 antibody (RLP30 serves as negative control). n = 4 (mean ± SD); n.s, no significance and ***p < 0.001 by student's *t*-test. **C** ChIP-PCR showed that the H3K9me2 level of APC promoter. Hep3B or Huhl in 2B was treated with UNC0642 at a dose of 5.0 μM for 24 h, and then 1.0 × 10^7^ cells were subjected to ChIP using the H3K9me2 antibody (IgG control is GFP antibody). n = 4 (mean ± SD); n.s, no significance and ***p < 0.001 by student's *t*-test. **D** ChIP-PCR showed that PolII directly binds the APC promoter in Hep3B or Huhl cells. Cells and treatment were the same as 5C. 1.0 × 10^7^ cells were subjected to ChIP using the PolII antibody (IgG control is GFP antibody). n = 4 (mean ± SD); n.s, no significance, *p < 0.05 and **p < 0.01 by student's *t*-test. **E** RT-qPCR showed APC expression in Hep3B and Huh1 cells. Cells and treatment were the same as 5C. n = 6 (mean ± SD); ***p < 0.001 by student's *t*-test. **F** The APC protein level in cells was determined by Western blot. Hep3B and Huh1 were treated with UNC0642 at the indicated concentrations. n = 6 (mean ± SD); ***p < 0.001 by one-way ANOVA

To investigate the mechanism by which EHMT2 regulates Wnt–β-catenin signaling activity, we analyzed the ChIP-seq data using EHMT2 antibody in HepG2 cells from (Encyclopedia of DNA Elements) ENCODE database to identify its putative target genes. Intriguingly, a wide EHMT2 bind peak was found around the promoter region of *APC*, a tumor suppressor that promotes the rapid degradation of β-catenin to negatively regulate Wnt–β-catenin signaling (Fig. 5A, circled by a red rectangle). Therefore, we designed primers that specifically bind around the EHMT2 bind peak to carry out ChIP-PCR to confirm these observations. As shown in Fig. 5B, our results indeed displayed that EHMT2 is specifically bound to *APC*'s promoter both in Hep3B and Huh1 cells (Fig. 5B). To demonstrate the EHMT2 reaches *APC*'s promoter to execute functions, we treated cells with UNC0642 and then detected the H3K9me2 level of the promoter by ChIP-PCR assay. Our data showed that the *APC* promoter's H3K9me2 level was reduced after UNC0642 treatment (Fig. 5C) Moreover, we found that, in Hep3B and Huh1 cells, the binding of polymerase II (PolII) on *APC*'s promoter was slightly but significantly enhanced by UNC0642 treatment (Fig. 5D). All the lines of cues suggest that EHMT2 functionally binds to *APC*'s promoter. More direct evidence indicated that UNC0642 treatment dramatically up-regulated *APC* mRNA level, which was largely abrogated by deletion of EHTM2 in Hep3B and Huh1 cells (Fig. 5E). Besides, APC protein levels also were induced by UNC0642 treatment in a dose-dependent manner (Fig. 5F). Taken together, our data demonstrate that EHMT2 directly binds to the *APC* promoter to suppress its expression by mediating H3K9me2.

### APC mediates EHMT2's oncogenic functions in HCC

**Fig. 6:**
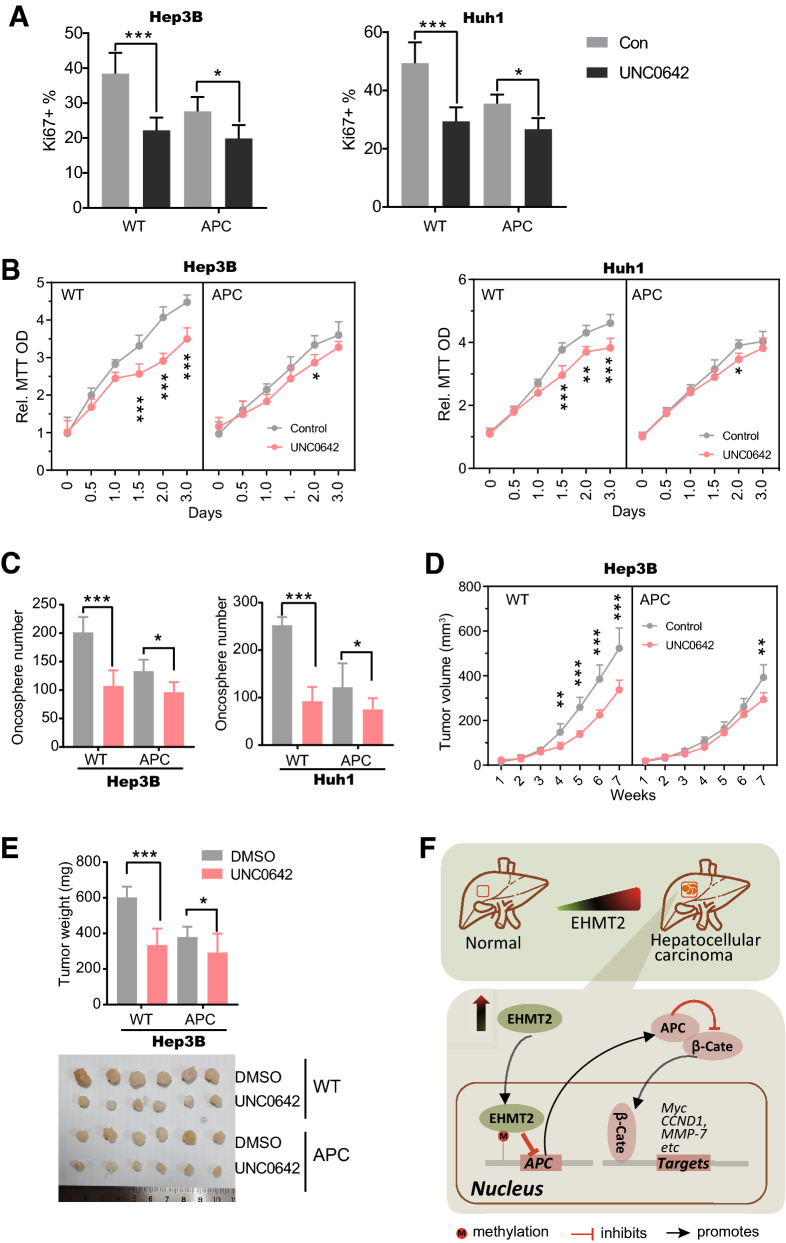
APC mediates EHMT2's oncogenic functions in HCC. **A** Cell proliferation was determined by Ki67 staining. Hep3B and Huh1 cells were stably transfected with APC and treated with UNC0642 at a dose of 1.0 μM for 24 h. The nucleus of harvested cells was isolated. n = 6 (mean ± SD); n = 6 (mean ± SD); *p < 0.05 and ***p < 0.001 by student's *t*-test. **B** MTT assay determined the cell growth curve of Hep3B and Huh1 cells. Cells were treated with UNC0642 at a dose of 1.0 μM for 3 days. n = 6 (mean ± SD); *p < 0.05, **p < 0.01 and ***p < 0.001 by two-way ANOVA followed by post hoc Bonferroni multiple comparisons. **C** Soft agar assay determined the anchorage-independent growth ability of Hep3B and Huh1 cells. APC-overexpressed Hep3B and Huh1 cells were treated with UNC0642 at a dose of 1.0 μM with an interval of 3 days. n = 6 (mean ± SD); *p < 0.05 and ***p < 0.001 by student's *t*-test. **D** Tumor growth curve of Hep3B xenograft assay. APC-overexpressed Hep3B cells in 6A were used. Animals were administrated treated with UNC0642 at a dose of 1 mg/kg body weight via intraperitoneal with an interval of 3 days. n = 6 (mean ± SD); *p < 0.05, **p < 0.01 and ***p < 0.001 by two-way ANOVA followed by post hoc Bonferroni multiple comparisons. **E** Final tumor weight of Hep3B xenograft assay in 6D. The bottom picture is a representative of final tumors. n = 6 (mean ± SD); *p < 0.05 and ***p < 0.001 by student's *t*-test. **F** A working model shows the roles of EHMT2 in the pathogenesis of HCC. β-cate, β-catenin

It was well documented that APC involves the pathogenesis of various cancers including colorectal cancers, pancreatic duct adenocarcinoma, oral cancers, breast cancers [38–41]. To explore its possible association between EHMT2 and APC in HCC, we stably transfected *APC* into Hep3B and Huh1 cells using a lentivirus vector. In the Ki67 staining assay, we found that the inhibitory effects of UNC0642 on cell proliferation were significantly weakened by overexpression of APC, indicated by the UNC0642-induced reduction of Ki67^+^ percentage was obviously decreased in APC-overexpressed Hep3B and Huh1 cells when compared with wild type ones (Fig. 6A). Consistently, the growth curve by MTT assay also showed that the UNC0642-induced cell growth suppression was substantially alleviated in APC-overexpressed Hep3B and Huh1 cells (Fig. 6B). Furthermore, in the soft agar assay, we observed that UNC0642-induced inhibition of tumorsphere formation was partially relieved in APC-overexpressed Hep3B and Huh1 cells (Fig. 6C). These observations indicate that the oncogenic effects of EHMT2 largely are medicated by APC in vitro. We also carried out a xenograft assay using APC-overexpressed Hep3B and Huh1 cells to investigate the association between EHMT2 and APC in tumor growth in vivo. We found that UNC0642 treatment significantly suppressed tumor growth both in Hep3B and Huh1 cells (Fig. 6D and Additional file 1: Figure S4A). These effects were largely reversed by overexpression of APC in cells, shown by the UNC0642-induced difference in tumor growth were observed at the 7th week in the overexpression group instead of the 4^th^ week in the control group (Fig. 6D and Additional file 1: Figure S4A). Likely, the final tumor weighs of xenograft assay also displayed consistent results with tumor growth curve both in APC-overexpressed Hep3B and Huh1 cells (Fig. 6E, and Additional file 1: Figure S6B). Our observations collectively evidence that APC largely mediates EHMT2's oncogenic functions in HCC both in vitro and in vivo.

## Discussion

Previously, alteration of *EHMT2* expression has been frequently observed in various human cancer types such as ovarian cancer, melanoma, esophageal squamous cell carcinoma, aggressive lung cancer, brain cancer [25, 28, 42]. For instance, in primary melanoma, higher expression of *EHMT2* also was found when compared to normal skin samples, and knocking down its expression via small interfering RNA significantly reduces cell viability, migration, and invasion in human M14 and A375 melanoma cell lines in vitro [36]. In our study, we observed that *EHMT2* expression in liver tumors was significantly 4–5 folds higher than their adjacent normal tissues both in mRNA and protein levels. Moreover, the high *EHMT2* expression was unfavorable for the prognosis outcome of HCC patients in overall and disease-free survival. In three different HCC subtypes proposed by Chaisaingmongkol et al., *EHMT2* expression (in mRNA level) still exhibited considerable discrimination (with HR > 2.1) in the overall survival of patients with HCC. Furthermore, we knocked out *EHMT2* expression using CRISPR/Cas9 in Hep3B and Huh1 cells and observed that the deletion of *EHMT2* restrained cell proliferation in vitro and tumor growth in vivo. All the results mentioned above demonstrated that the up-regulation of EHMT2 in liver cancer is functional. In fact, Wei et al. previously reported that *EHMT2* expression is upregulated in human HCC, and they considered that the alteration of *EHMT2* expression is attributed to miR-1 [43]. We also determined miR-1 expression in specimens of HCC patients but failed to detect a significant difference in expression level between tumor and normal tissues (data not shown). These data indicated that the mechanisms causing *EHMT2* expression alteration are quite complex, which needs further investigation.

In mouse models of acute myeloid leukemia, Lehnertz et al. established EHMT2 as a selective regulator of fast proliferating myeloid progenitors, indicated by the evidence that loss of EHMT2 significantly delays disease progression and reduces leukemia stem cell frequency by interacting with the leukemogenic transcription factor HoxA9 [27]. However, HoxA9 expression is too low to be detected both in liver normal and tumor tissues (data not shown) in our experiment. Recently, Ma and co-workers presented evidence that the histone-lysine methyltransferase EHMT2 is up-regulated in human cholangiocarcinoma, which enhances cell growth and invasiveness by epigenetically silencing the Hippo pathway kinase large tumor suppressor 2 (*LATS2*) [44]. We also analyzed *LATS2* expression using public data from TCGA, and unfortunately, no significant difference was observed in the mRNA level of *LATS2*. In 2017, Wei et al. identified *EHMT2* as a frequently upregulated histone methyltransferase in patients with HCC, which contributes to epigenetic silencing of tumor suppressor gene RARRES3 [43]. Hu et al. reported that EHMT2 protein interacted with Snail2 and histone deacetylases (HDACs) to form a complex to suppress E-cadherin (a hallmark of epithelial-mesenchymal transition EMT) transcription in HCC [45]. In our study, we demonstrated that EHMT2 is implicated in the regulation of Wnt–β-catenin signaling pathway in HCC by several lines of evidence: (1) EHMT2 directly binds to the promoter of *APC* in HepG2, Hep3B and Huh1; (2) EHMT2 mediates H3K9me2 of *APC* promotor; (3) EHMT2 promotes the expression of *APC* both in mRNA and protein levels; (4) the functions of EHMT2 are largely abolished by deletion of APC in Hep3B and Huh1 cells. Therefore, we considered that EHMT2 is versatile in the pathogenesis of HCC.

Over the past decade, the anti-tumor effects of EHMT2 inhibitors have been extensively investigated by independent groups. In 2018, Zhang et al. reported that UNC0638, a selective EHMT2 inhibitor significantly inhibited tumor growth of A549, H1299, and H1975 cell lines [23]. Cao et al. found that UNC0642 exhibits excellent anticancer effects with low cytotoxicity against urinary bladder cancer cells including T24, J82, and 5637 both in vitro and in vivo [31]. Also, Dong et al. demonstrated that the UNC0642 decreased cell viability and increased apoptosis of melanoma cells including M14 and A375 cell lines with minimal cell toxicity and good in vivo pharmacokinetic characteristics [36]. In this study, our findings showed that UNC0642 treatment also inhibited the H3K9me2 level and suppressed cell proliferation in vitro and tumor growth in vivo in Hep3B and Huh1 cells. Moreover, all these observations were largely abolished by the deletion of *EHMT2* from cells, suggesting UNC0642 achieves its anti-tumor effects in an EHMT2 dependent manner. Besides, we demonstrated that UNC0642 inhibits the Wnt–β-catenin signaling pathway by several lines of evidence: 1) UNC0642 decreases Wnt3a-induced TOP/FOP luciferase activity and the expression of Wnt–β-catenin signaling pathway including *Myc, CCND1* and *MMP-7*; 2) UNC0642 reduces the H3K9me2 level of *APC* promoter; 3) UNC0642 treatment inhibits PolII binding to *APC* promoter; 4) overexpression of APC from cells largely abolished the effects of UNC0642 treatment. In another line, UNC0642 is also an inhibitor of EHMT1, a homolog of EHMT2. In our experiment, we also observed that UNC0642 treatment is a little more powerful than deletion of *EHMT2* on inhibiting cell proliferation, probably because UNC0642 treatment simultaneously suppresses the activity of EHMT1 and 2 in cells. Meanwhile, we noticed that the expression level of *EHMT1* is around 1/3 of *EHMT2* in mRNA, which also fails to alter in HCC tissues (data not shown). These observations suggest that *EHMT2* rather than *EHMT1* plays a dominant role in the pathogenesis of HCC. Altogether, we consider that UNC0642 possesses anti-tumor functions on HCC mainly by targeting EHMT2.

## Conclusion

In summary, we observed that the mRNA level of *EHMT2* in HCC elevated significantly when compared with the normal controls of specimens from the public database and HCC patients we collected (Fig. 6F). The elevation of *EHMT2* expression is required for HCC tumorigenesis and contributes to the activation of the Wnt–β-catenin pathway by epigenetically silencing *APC* expression (Fig. 6F). UNC0642 exhibits anti-tumorigenesis effects in HCC both in vitro and in vivo by targeting *EHMT2* in Hep3B and Huh1 cells. Altogether, our observations emphasize that the EHMT2-APC axis is a contributor to Wnt–β-catenin pathway activation in HCC, and UNC0642 may be a potential candidate for target drug for HCC treatment.

## Supplementary Information

**Additional file 1: Figure S1.** High EHMT2 expression is unfavorable for patients with HCC. A, B EHMT2-based overall and disease-free survival analysis of patients with iClust1/2/3 HCC. In OS and DFS analysis, EHMT2 expression data from TCGA were used, and the median of EHMT2 expression served as the cutoff. High EHMT2, expression level more than median; Low EHMT2, expression level less than the median. A log-rank test was used for the hypothesis test. **Figure S2.** EHMT2 is required for the tumorigenesis of HCC. Tumor growth curve of Hep3B and Huh1 in a xenograft assay. Three EHMT2−/− single clones of Hep3B and Huh1 in 2A were pooled together for xenograft assay. n = 6 (mean ± SD); *p < 0.05, **p < 0.01 and ***p < 0.001 by two-way ANOVA followed by post hoc Bonferroni multiple comparisons. **Figure S3.** EHMT2 inhibitor exhibits anti-tumorigenesis effects in HCC. Tumor growth curve of Hep3B xenograft assay. Cells in 3B were used. UNC0642 treated animals with a dose of 5 mg/kg via intraperitoneal injection with an interval of 3 days. n = 6 (mean ± SD); *p < 0.05 and ***p < 0.001 by two-way ANOVA followed by post hoc Bonferroni multiple comparisons. **Figure S4.** APC mediates EHMT2's oncogenic functions in HCC. A. Tumor growth curve of Huh1 xenograft assay. APC-overexpressed Huh1 cells in 6A were used. Animals were administrated treated with UNC0642 at a dose of 1 mg/kg body weight via intraperitoneal with an interval of 3 days. n = 6 (mean ± SD); *p < 0.05, **p < 0.01 and ***p < 0.001 by two-way ANOVA followed by post hoc Bonferroni multiple comparisons. B. Final tumor weight of Huh1 xenograft assay in S4A. The bottom picture is a representative of final tumors. n = 6 (mean ± SD); *p < 0.05 and ***p < 0.001 by student's t-test.

## Data Availability

ChIP-seq data using EHMT2 antibody are available in GSE170681 at https://www.ncbi.nlm.nih.gov/geo/query/acc.cgi?acc=GSE170681 [doi:10.17989/ENCSR376FMN], reference number [PMID: 27528022].
