# Supplementary figures and images for: EHMT2 promotes the pathogenesis of hepatocellular carcinoma by epigenetically silencing APC expression
Source: Cell Biosci. 2021 Aug 3;11:152. doi: 10.1186/s13578-021-00663-9 (PMC8335875; doi:10.1186/s13578-021-00663-9)

Fig S1. Related to Fig 1

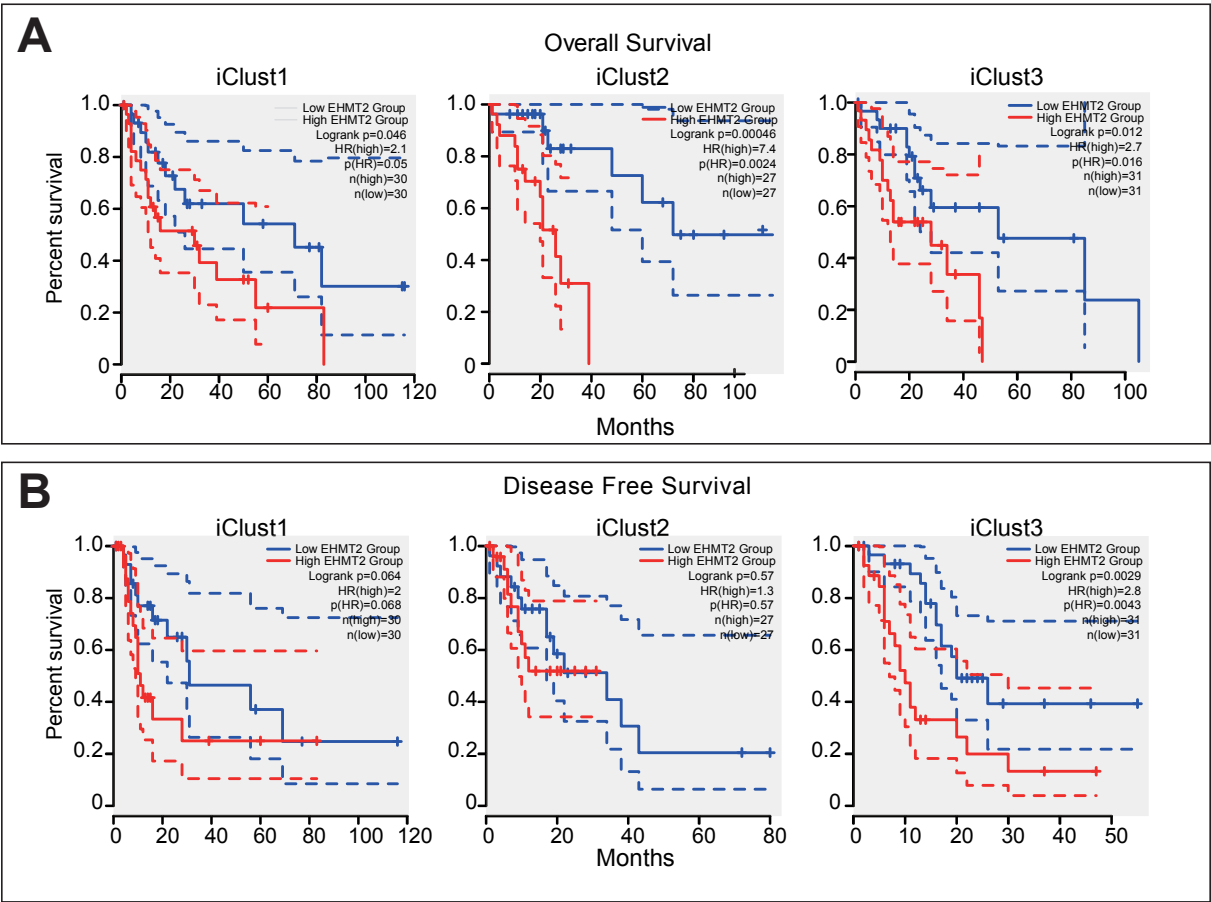

Fig S2. Related to Fig 2

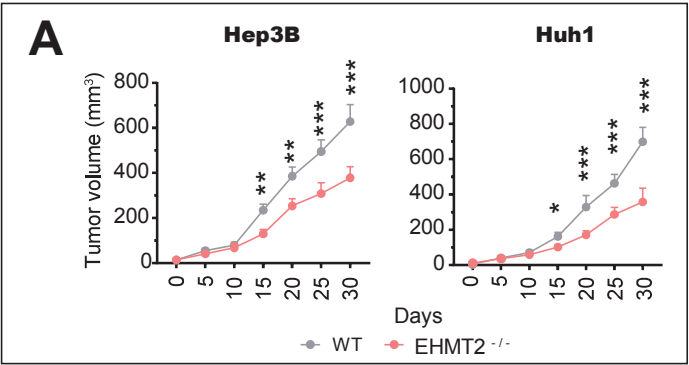

Fig S3. Related to Fig. 3

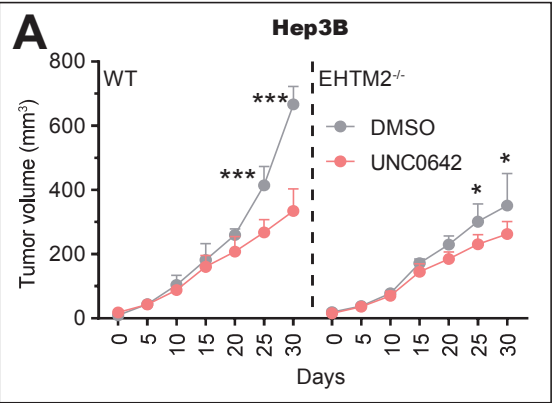

Fig S4. Related to Fig. 6

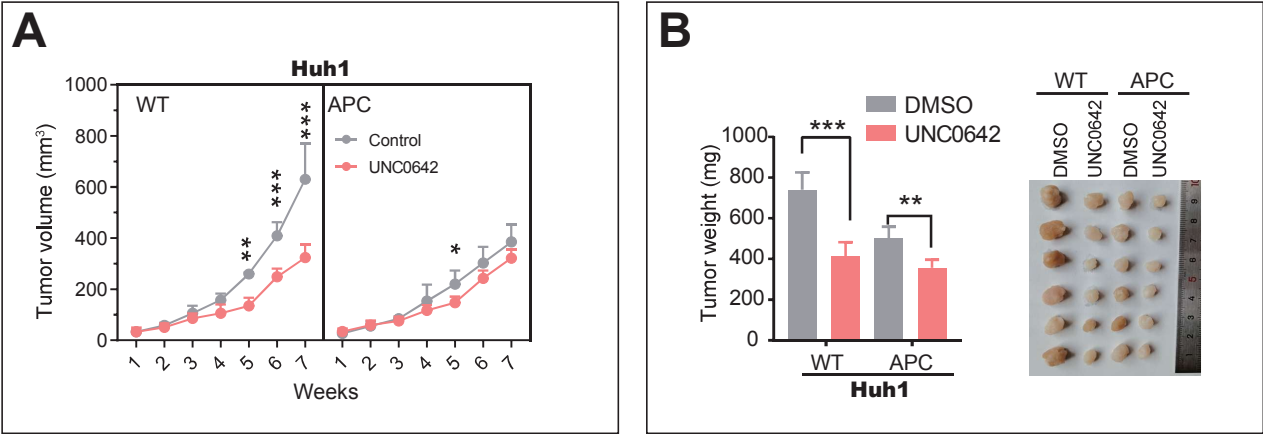

Supplement: Supplementary file 1 — Additional file 1: Figure S1. High EHMT2 expression is unfavorable for patients with HCC. A, B EHMT2-based overall and disease-free survival analysis of patients with iClust1/2/3 HCC. In OS and DFS analysis, EHMT2 expression data from TCGA were used, and the median of EHMT2 expression served as the cutoff. High EHMT2, expression level more than median; Low EHMT2, expression level less than the median. A log-rank test was used for the hypothesis test. Figure S2. EHMT2 is required for the tumorigenesis of HCC. Tumor growth curve of Hep3B and Huh1 in a xenograft assay. Three EHMT2−/− single clones of Hep3B and Huh1 in 2A were pooled together for xenograft assay. n = 6 (mean ± SD); *p < 0.05, **p < 0.01 and ***p < 0.001 by two-way ANOVA followed by post hoc Bonferroni multiple comparisons. Figure S3. EHMT2 inhibitor exhibits anti-tumorigenesis effects in HCC. Tumor growth curve of Hep3B xenograft assay. Cells in 3B were used. UNC0642 treated animals with a dose of 5 mg/kg via intraperitoneal injection with an interval of 3 days. n = 6 (mean ± SD); *p < 0.05 and ***p < 0.001 by two-way ANOVA followed by post hoc Bonferroni multiple comparisons. Figure S4. APC mediates EHMT2's oncogenic functions in HCC. A. Tumor growth curve of Huh1 xenograft assay. APC-overexpressed Huh1 cells in 6A were used. Animals were administrated treated with UNC0642 at a dose of 1 mg/kg body weight via intraperitoneal with an interval of 3 days. n = 6 (mean ± SD); *p < 0.05, **p < 0.01 and ***p < 0.001 by two-way ANOVA followed by post hoc Bonferroni multiple comparisons. B. Final tumor weight of Huh1 xenograft assay in S4A. The bottom picture is a representative of final tumors. n = 6 (mean ± SD); *p < 0.05 and ***p < 0.001 by student's t-test. [file 13578_2021_663_MOESM1_ESM.pdf]
